# Genome-wide identification of the class III peroxidase gene family and its association with fruit rind cracking in *Cucumis melo*

**DOI:** 10.3389/fpls.2025.1706618

**Published:** 2026-01-21

**Authors:** Yanping Hu, Tingting Zhang, Yushan Wang, Chongchong Wang, Baibi Zhu, Feng Wang, Yisong Chen, Min Wang, Yang Zhou

**Affiliations:** 1 The Institute of Vegetables, Hainan Academy of Agricultural Sciences, Key Laboratory of Vegetable Biology of Hainan Province, Hainan Vegetable Breeding Engineering Technology Research Center, Haikou, China; 2 Key Laboratory for Quality Regulation of Tropical Horticultural Crops of Hainan Province, School of Tropical Agriculture and Forestry (School of Agricultural and Rural Affairs, School of Rural Revitalization), Hainan University, Haikou, China; 3 Sanya Institute, Hainan Academy of Agricultural Sciences, Sanya, China; 4 Xiangyang Academy of Agricultural Sciences, Xiangyang, China

**Keywords:** fruit cracking, gene expression, lignin accumulation, melon, PRX gene family

## Abstract

**Introduction:**

Class III peroxidase (PRX) functions as a pivotal enzyme in lignin polymerization and participates in the regulation of cell wall hardening and elongation. Nevertheless, comprehensive investigations on PRX involvement in the rind cracking of melon (*Cucumis melo*) remain absent.

**Methods:**

In this study, melon was used as experimental material. Physiological analyses were performed to compare peroxidase activity and lignin accumulation between cracking-susceptible and resistant cultivars, as well as between cracked and non-cracked rinds. Genome-wide identification, phylogenetic analysis, chromosome localization, collinearity analysis, and *cis*-acting element prediction were conducted to characterize the melon PRX gene family. Transcriptome sequencing was used to analyze *CmPRX* expression patterns across different rind types, and quantitative real-time polymerase chain reaction (qRT-PCR) was performed for validation. Protein-protein interaction networks were predicted to explore the functional associations of candidate genes.

**Results and discussion:**

Peroxidase activity and lignin accumulation were significantly higher in cracking-susceptible cultivars compared to cracking-resistant cultivars, with cracked rinds displaying elevated levels relative to intact rinds. Sixty-four *PRX* genes were identified in the melon genome, and phylogenetic analysis categorized them into six subgroups. The *CmPRX* genes were unevenly distributed across 12 chromosomes, and collinearity analysis uncovered eight duplicated gene pairs within the melon genome. Comparative synteny analysis revealed that the number of collinear *PRX* gene pairs between melon and other Cucurbitaceae species, specially cucumber and watermelon, was greater than that observed with the more distantly related *Arabidopsis*. Promoter *cis-*acting element examination revealed that the 64 *CmPRX* genes harbored 25 classes of elements associated with hormones, stress responses, and growth and development. Transcriptome data from melon rinds revealed that the *CmPRX* genes could be clustered into six groups based on expression patterns across different rind types. Among these, *CmPRX* genes in clusters 1 and 6 exhibited higher transcript levels in cracked rinds compared to non-cracked rinds. Moreover, quantitative real-time polymerase chain reaction analyses confirmed that *CmPRX39*, *CmPRX48*, and *CmPRX51* were expressed at significantly elevated levels in cracked rinds compared with those of non-cracked rinds. Protein interaction network prediction showed that these three candidate genes interacted with multiple proteins involved in the lignin synthesis pathway, suggesting their potential regulatory roles in rind cracking of melon through mediating lignin polymerization. These findings identified candidate genes influencing rind cracking in melon, thereby offering potential molecular targets for the breeding of cracking-resistant cultivars.

## Introduction

Fruit cracking represents a widespread and deleterious physiological disorder that markedly compromises fruit quality. The occurrence of fruit cracking results in diminished visual appeal and heightened vulnerability to pathogen invasion, thereby impeding the sustainable advancement of the fruit industry (Bruggenwirth and Knoche, 2017; Schumann et al., 2019; Wang et al., 2021; Santos et al., 2023; Hu et al., 2024). In China, the predominant commercial cultivars of melon (*Cucumis melo*) generally exhibit limited resistance to rind cracking. Melon rind cracking predominantly manifests during the late phase of fruit enlargement. Fruits experiencing rind cracking not only possess a reduced shelf life but also incur elevated transportation and storage expenses, ultimately causing substantial economic losses for producers (Qi et al., 2015).

Fruit cracking is governed by multiple factors, including genetic background, environmental stresses, cultivation practices, and postharvest storage conditions (Simon, 2006; Khadivi-Khub, 2015; Correia et al., 2018; Wang et al., 2021). When the internal pressure exerted on fruit tissues surpasses the mechanical resistance of the cell walls and cuticles, fissures develop on the epidermis (Bruggenwirth and Knoche, 2017). Among these diverse determinants, dynamic alterations in cell wall constituents constitute the central factor dictating the mechanical robustness of the pericarp. As a principal component of secondary cell walls, lignin deposition leads to lignification, restricts cell elongation, and reduces cell wall extensibility, thereby influencing fruit cracking (Musel et al., 1997). Previous investigations have demonstrated that lignin levels in cracking-susceptible pepper fruits increase during the cracking process, and severely cracked fruits exhibit significantly higher lignin levels compared with non-cracked pepper fruits (Liu et al., 2022). Similarly, lignin content in the pericarp of cracking-susceptible litchi varieties is greater than that of cracking-resistant varieties (Wang et al., 2019). Lignin biosynthesis predominantly originates from three hydroxycinnamic alcohol precursors: p-coumaryl alcohol, coniferyl alcohol, and sinapyl alcohol. These precursors give rise to H-lignin monomers (p-hydroxyphenyl), G-lignin monomers (guaiacyl), and S-lignin monomers (syringyl), respectively. Peroxidases (POD) function as pivotal enzymes in catalyzing the polymerization of lignin monomers into complex lignin polymers (Barros et al., 2015; Kumar et al., 2016).

POD constitutes a class of enzymes widely distributed in living organisms, catalyzing oxidation–reduction reactions in which hydrogen peroxide serves as an electron acceptor and diverse compounds act as electron donors (Passardi et al., 2005). On the basis of protein sequence and structural features, POD is categorized into non-heme POD and heme POD, with the latter further divided into animal heme POD and non-animal heme POD. Non-animal heme POD is subdivided into three classes: Class I, Class II, and Class III peroxidases (PRX) (Almagro et al., 2009). PRXs are plant-specific oxidoreductases that constitute secretory POD derived from higher plants, engaging in diverse physiological processes and functioning as pivotal enzymes in maintaining intracellular redox balance and ensuring cellular homeostasis, thereby playing fundamental roles in plant growth, development, and stress adaptation (Vogelsang and Dietz, 2022; Shen et al., 2025). Functional studies of *PRX* genes in other plants have provided clues for their role in fruit cracking. Certain PRX destabilize plant cell wall integrity through the generation of hydroxyl radicals capable of cleaving load-bearing cell wall polysaccharides (Liszkay et al., 2004). In *Arabidopsis*, PRX9 and PRX40 maintain tapetum cell wall integrity through extensin cross-linking (Jacobowitz et al., 2019), while PEROXIDASE36 operates as a seed mucilage extrusion factor by regulating the degradation of the outer cell wall of *Arabidopsis* outermost integument cells (Kunieda et al., 2013). In pepper fruit development, nine *POD* genes were identified with continuously elevated expression from non-cracked fruits to severely cracked fruits, in which higher lignin accumulation was observed in cracked fruits (Liu et al., 2022). These studies indicate that *PRX* genes play conserved roles in cell wall metabolism and lignin accumulation, but their function in melon rind cracking remains unclear.

Analysis of cell wall components in the rind of thick-skinned melon with varying cracking resistance demonstrated that cellulose and hemicellulose levels were markedly higher in extremely cracking-resistant fruits than in extremely cracking-susceptible types, whereas POD activity in the rind of extremely cracking-susceptible melon was significantly greater than that of extremely cracking-resistant fruits (Fan et al., 2023). Transcriptome sequencing of rinds from cracking-resistant and cracking-susceptible melon varieties revealed that *POD* gene expression exhibited a significant positive correlation with rind cracking rate (Fan et al., 2025). These observations indicate that POD-mediated modification of cell walls may constitute a major mechanism governing fruit rind cracking in melon. Nevertheless, systematic investigations into its functional role in melon rind cracking remain limited. Previous studies have linked PRX-mediated lignin polymerization to fruit cracking in pepper (Liu et al., 2022) and litchi (Wang et al., 2019), but the molecular mechanisms underlying PRX function in cucurbit-specific fruit rind development and cracking remain unclear. To address this gap, bioinformatics approaches were employed in the present study to identify PRX gene family members at the whole-genome level in melon, and their physicochemical characteristics, evolutionary relationships, gene architectures, chromosomal distributions, and promoter *cis*-acting elements were comprehensively analyzed. In combination with transcriptome data from rinds of cracking-resistant and cracking-susceptible melon, the expression profiles of *CmPRX* genes were examined, candidate genes potentially associated with fruit rind cracking were identified, and their differential expression was validated using reverse transcription quantitative polymerase chain reaction (RT-qPCR). By identifying the complete *CmPRX* gene family in melon genomes and elucidating its association with fruit peel cracking, this study provides new insights into the genetic improvement of cucurbit crops.

## Materials and methods

### Determination of lignin content and POD activity in melon rinds

During the late fruit expansion stage of melon, fruit rind cracking rates of different melon types were quantitatively assessed in the field (cracking rate = number of cracking fruits/total number of examined fruits × 100%). Rind samples from melon types exhibiting varying degrees of cracking resistance were collected, and lignin contents (No. G0708W) together with POD enzymatic activity (No. G0107W) were measured using commercial reagent kits supplied by Suzhou Grace Biotechnology Co., Ltd.

### Identification of the PRX gene family in melon

The hidden Markov model (HMM) profile of the PRX domain (PF00141) was retrieved from the Pfam (http://pfam.xfam.org/) database, and hmmsearch was executed in TBtools (Chen et al., 2020) with the HMM file to query the melon genome. Meanwhile, AtPRX and ClPRX protein sequences were obtained from the *Arabidopsis thaliana* and watermelon (*Citrullus lanatus*) (Yang et al., 2022) genome databases, respectively, and BLAST searches (E < 1 × 10^−5^) were carried out against the melon genome using TBtools. Candidate protein and CDS sequences were extracted from the melon genome files with TBtools. The resulting candidate protein sequences were subsequently aligned to the Pfam, SMART (https://smart.embl.de/smart/change_mode.cgi), and CDD (https://www.ncbi.nlm.nih.gov/cdd/) databases for domain prediction. Proteins containing PRX domains were ultimately recognized as members of the melon PRX gene family.

### Analysis of physicochemical properties and evolutionary relationships of CmPRX proteins

The amino acid length, isoelectric point, and molecular weight of CmPRX proteins were evaluated using the ExPASy (https://web.expasy.org/protparam/) online platform. Multiple sequence alignment of PRX domain sequences from melon, *Arabidopsis*, tobacco (*Nicotiana tabacum*) (Cheng et al., 2022), cucumber (*Cucumis sativus*) (Luo et al., 2024), and watermelon was conducted with MEGA X, and a phylogenetic tree was generated by the Maximum-Likelihood method with the bootstrap value set at 1,000, whereas other parameters were maintained at default settings. Then the phylogenetic tree was visualized and enhanced using the EvolView online tool (Evolview v3; https://evolgenius.info/evolview-v2) (Subramanian et al., 2019).

### Analysis of conserved motifs and gene structure of CmPRX proteins

Conserved motifs of CmPRX proteins were identified using the MEME (http://meme-suite.org/) online suite with the maximum motif number set to 10. Visualization of gene structures was subsequently conducted in TBtools with the melon gene structure annotation GFF file, genomic sequences, and CDS sequences.

### Chromosomal localization and collinearity analysis of *CmPRX* genes

The melon genome annotation file was retrieved, and chromosomal localization of *CmPRX* genes within the melon genome was analyzed using TBtools. To examine collinearity relationships of *CmPRX* genes, chromosome lengths were obtained through the Fasta Stat function in TBtools. Positional information of *CmPRX* genes was extracted with the GXF Gene Position & Info Extract function, whereas homologous duplication information within the genome was obtained using the One Step MCScanX function. Finally, chromosomal collinearity maps were generated with Advanced Circos. Meanwhile, Syntenic maps were generated using Circos software to illustrate the synteny relationships among the orthologous *PRX* genes derived from melon and other plant species.

### Analysis of promoter *cis*-acting elements

Upstream sequences of 2,000 bp from the start codon of *CmPRX* genes were extracted as promoter regions using TBtools. *Cis*-acting elements were identified through the PlantCARE (http://bioinformatics.psb.ugent.be/webtools/plantcare/html/) online platform, and the results were subsequently visualized with TBtools.

### Gene expression analysis

FPKM (Fragments Per Kilobase of exon model per Million mapped fragments) values of *CmPRX* genes were derived from transcriptome data of fruit rinds from cracking-resistant and cracking-susceptible varieties obtained in our lab (SRA No.: SRP466450). A heatmap representing the differential expression patterns of *CmPRX* genes was generated via TBtools. Gene expression clustering across different rind samples was subsequently performed using the *K*-means analysis function in R software (version stats 4.2.0) with default parameters, as available on the Metware Cloud Platform (https://cloud.metware.cn).

On the basis of RNA sequencing (RNA-seq) results, differentially expressed genes were validated through RT-qPCR. The *CmActin* gene served as the internal reference, and primer sequences for selected genes and the reference gene are provided in **Supplementary Table S1**. RT-qPCR amplification was performed with ChamQ™ Universal SYBR qPCR Master Mix (Vazyme, Q711-03). The RT-qPCR reaction system and program followed the method of Hu et al. (2024). Three biological replicates were performed for each sample, with three technical replicates for each biological replicate. Relative gene expression levels were calculated using the 2^−△△CT^ method (Hu et al., 2023).

To further examine expression patterns of *CmPRX* genes in rinds of different melon varieties, samples from multiple melon types were collected. RNA was extracted using a plant total RNA extraction kit (TianGen Biotech (Beijing) Co., Ltd., DP437), and first-strand cDNA was synthesized with a reverse transcription kit (TaKaRa (Dalian), RR047A). RT-qPCR was then conducted using ChamQ™ Universal SYBR qPCR Master Mix, with all procedures executed per the supplier’s protocols.

### Analysis of the interaction network of CmPRX proteins

To better understand the interaction network of CmPRX proteins, we utilized STRING software (version 12.0, https://cn.string-db.org/) to analyze the interactions between CmPRX proteins and other proteins (Szklarczyk et al., 2023). The ‘Multiple proteins by sequence’ method was employed, with ‘*Cucumis melo*’ selected as the organism for analysis. The amino acid sequences of three proteins, CmPRX39, CmPRX48, and CmPRX51, were used to query the STRING database via the BLASTP method with default parameters, leading to the establishment of the protein interaction network chart.

### Statistical analysis

All experiments were performed in three biological replicates unless otherwise stated. Statistical analysis was carried out utilizing the SPSS 20 software, and statistical differences were assessed utilizing one way analysis of variance (ANOVA). Data are expressed as means with standard errors, and p < 0.05 was regarded as statistically significant.

## Results

### Analysis of lignin content and POD activity in different melon germplasms

**Figure 1:**
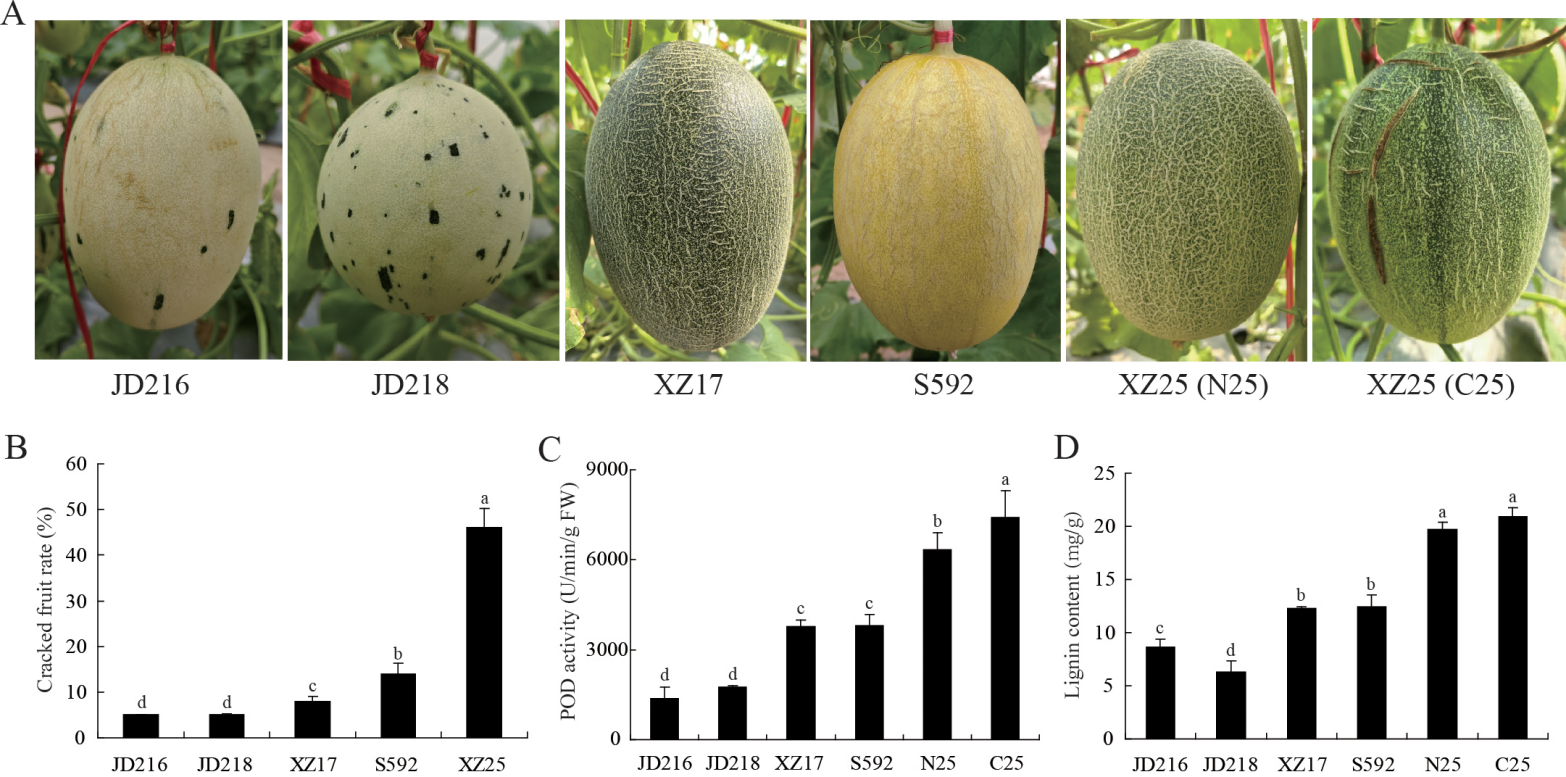
Determination of physiological indicators in the rinds of different melon germplasms. **(A)** Phenotypic characteristics of different melon germplasms. **(B)** Field incidence of fruit cracking in different melon germplasms. **(C)** Measurement of peroxidase (POD) enzyme activity in the rinds of various melon types. **(D)** Quantification of lignin content in the rinds of different melon varieties. Different lowercase letters indicate statistically significant differences at P < 0.05.

Field investigations of fruit cracking were carried out on five *Cucumis melo* varieties (‘JD216’, ‘JD218’, ‘XZ17’, ‘S592’, ‘XZ25’) (**Figure 1A**). The cracking rate of ‘XZ25’ reached 46%, which was markedly higher than that observed in the other four *Cucumis melo* varieties (**Figure 1B**). Rinds were collected from different cracking-resistant melon varieties, and both non-cracked (N25) and cracked rinds (C25) were sampled from ‘XZ25’ (**Figure 1A**). Their POD activities were subsequently measured. POD activity in the rinds exhibited an increasing trend in parallel with the rise in cracking rate (**Figure 1C**). Lignin content analysis of the various rinds revealed that cracking-susceptible varieties displayed significantly higher lignin levels compared with cracking-resistant varieties (**Figure 1D**). These findings suggested that POD may be involved in the fruit rind cracking process of melon through the regulation of lignin polymerization.

### Identification and physicochemical property analysis of the melon PRX gene family

To systematically examine the potential role of *POD* genes in rind cracking of melon, the PRX gene family was identified. In total, 89 candidate proteins were initially retrieved from the melon genome using HMM files through hmmsearch in TBtools, while 76 additional candidates were obtained by blasting *Arabidopsis* and watermelon PRX protein sequences against the melon genome database. After domain verification through Pfam, SMART, and CDD databases, together with manual elimination of redundant sequences, 64 members of the *CmPRX* gene family were ultimately identified. According to their chromosomal locations, these 64 *CmPRX* genes were designated sequentially as *CmPRX1–64*. Analysis of physicochemical properties demonstrated that the amino acid lengths of the CmPRX proteins ranged from 263 to 381, molecular weights from 28.91 to 42.65 kD, and isoelectric points from 4.53 to 9.50 (**Table 1**).

**Table 1:** Information of melon PRX gene family.

Gene name	Gene ID	No. of chromosome	Genome location	Length of amino acid	Molecular weight (kDa)	Isoelectric point	Group
CmPRX1	MELO3C018655	chr1	1820720…1823165	328	35.78	8.11	F
CmPRX2	MELO3C018656	chr1	1824185…1826354	328	35.40	8.66	F
CmPRX3	MELO3C018672	chr1	1986983…1993071	324	34.97	6.11	F
CmPRX4	MELO3C018719	chr1	2350805…2352997	314	34.04	8.63	A
CmPRX5	MELO3C018804	chr1	2918898…2920926	328	35.83	9.19	E
CmPRX6	MELO3C012610	chr1	19955395…19957071	381	42.65	5.61	G
CmPRX7	MELO3C023617	chr1	31788399…31789578	328	35.51	8.06	C
CmPRX8	MELO3C023615	chr1	31802854…31804643	331	35.88	5.43	C
CmPRX9	MELO3C023613	chr1	31815213…31816862	315	33.99	9.29	C
CmPRX10	MELO3C023612	chr1	31831951…31833650	338	37.15	8.33	C
CmPRX11	MELO3C015445	chr2	1771025…1772770	315	34.27	8.4	A
CmPRX12	MELO3C010344	chr2	16667831…16670541	322	35.60	5.81	F
CmPRX13	MELO3C017120	chr2	24961375…24962579	327	35.64	8.47	E
CmPRX14	MELO3C008185	chr3	1684159…1686173	331	35.62	5.41	E
CmPRX15	MELO3C008187	chr3	1718168…1720413	322	34.84	8.03	E
CmPRX16	MELO3C008188	chr3	1731814…1733860	325	34.78	6.87	E
CmPRX17	MELO3C019994	chr3	19802277…19804028	324	34.38	8.82	A
CmPRX18	MELO3C011348	chr3	24496260…24497563	319	34.84	9.09	E
CmPRX19	MELO3C011261	chr3	25097894…25102295	335	36.65	7.61	H
CmPRX20	MELO3C003377	chr4	699105…700976	336	37.85	5.69	A
CmPRX21	MELO3C003628	chr4	2841630…2842769	263	28.91	9.12	E
CmPRX22	MELO3C009924	chr4	26911905…26913417	326	36.02	8.47	E
CmPRX23	MELO3C009329	chr4	31653384…31656429	325	36.56	8.03	G
CmPRX24	MELO3C014658	chr5	587370…598540	319	34.26	5.47	B
CmPRX25	MELO3C014657	chr5	604485…606030	345	37.11	6.01	B
CmPRX26	MELO3C014656	chr5	606910…608654	345	37.19	5.69	B
CmPRX27	MELO3C014655	chr5	614417…615763	329	36.67	5.92	B
CmPRX28	MELO3C014654	chr5	619413…620871	333	36.36	8.09	B
CmPRX29	MELO3C014653	chr5	622716…624211	331	35.68	5.71	B
CmPRX30	MELO3C014652	chr5	627289…628677	338	36.75	8.96	B
CmPRX31	MELO3C014651	chr5	633800…635376	335	35.74	4.58	B
CmPRX32	MELO3C014650	chr5	637124…639128	334	36.44	6.12	B
CmPRX33	MELO3C014252	chr5	4804512…4807240	331	37.27	6.19	G
CmPRX34	MELO3C006862	chr6	6794661…6796941	316	34.69	7.56	E
CmPRX35	MELO3C016943	chr7	948471…951250	327	34.92	5.32	E
CmPRX36	MELO3C016405	chr7	22661334…22663790	343	37.37	9.12	A
CmPRX37	MELO3C017603	chr7	23440473…23443748	336	37.73	6.54	A
CmPRX38	MELO3C007545	chr8	3515640…3517262	327	36.67	6.94	B
CmPRX39	MELO3C007618	chr8	4143953…4145523	329	36.08	9.3	E
CmPRX40	MELO3C007868	chr8	5876611…5878665	332	36.37	8.75	A
CmPRX41	MELO3C007935	chr8	6347138…6348627	318	34.56	9.06	E
CmPRX42	MELO3C003275	chr8	32117993…32120865	314	34.69	8.41	E
CmPRX43	MELO3C021513	chr9	2895061…2896164	319	35.06	8.35	E
CmPRX44	MELO3C005456	chr9	20602414…20604258	337	35.93	4.53	B
CmPRX45	MELO3C012183	chr10	2141672…2144503	293	32.55	5.48	A
CmPRX46	MELO3C022604	chr10	16021455…16022662	316	34.27	8.07	A
CmPRX47	MELO3C020841	chr11	3588825…3591189	336	36.64	8.85	E
CmPRX48	MELO3C021914	chr11	5192174…5194596	335	37.20	5.04	B
CmPRX49	MELO3C019239	chr11	9632763…9634386	329	36.12	7.03	E
CmPRX50	MELO3C026869	chr11	20923898…20932626	331	34.41	6.99	A
CmPRX51	MELO3C026870	chr11	20943336…20945039	326	35.19	9.43	A
CmPRX52	MELO3C019612	chr11	21749001…21751218	345	38.52	9.49	D
CmPRX53	MELO3C025681	chr11	24805556…24807090	325	35.43	9.15	A
CmPRX54	MELO3C025683	chr11	24837321…24838816	332	34.93	6.78	A
CmPRX55	MELO3C025684	chr11	24848416…24849761	320	35.01	8.81	A
CmPRX56	MELO3C021373	chr11	27074339…27076771	327	35.99	9.5	E
CmPRX57	MELO3C021297	chr11	27653467…27655069	268	29.19	6.88	C
CmPRX58	MELO3C021259	chr11	27982503…27983910	337	36.20	4.87	A
CmPRX59	MELO3C022435	chr11	30657229…30658981	340	38.24	9.36	G
CmPRX60	MELO3C020501	chr12	349146…351169	319	34.41	5.51	A
CmPRX61	MELO3C002632	chr12	21365018…21367877	347	38.08	9.24	E
CmPRX62	MELO3C002457	chr12	22733461…22735502	331	37.78	8.74	G
CmPRX63	MELO3C002391	chr12	23201934…23204297	343	38.19	5.84	D
CmPRX64	MELO3C002242	chr12	24207070…24208669	334	37.07	7.59	E

### Evolutionary relationship analysis of PRX proteins

**Figure 2:**
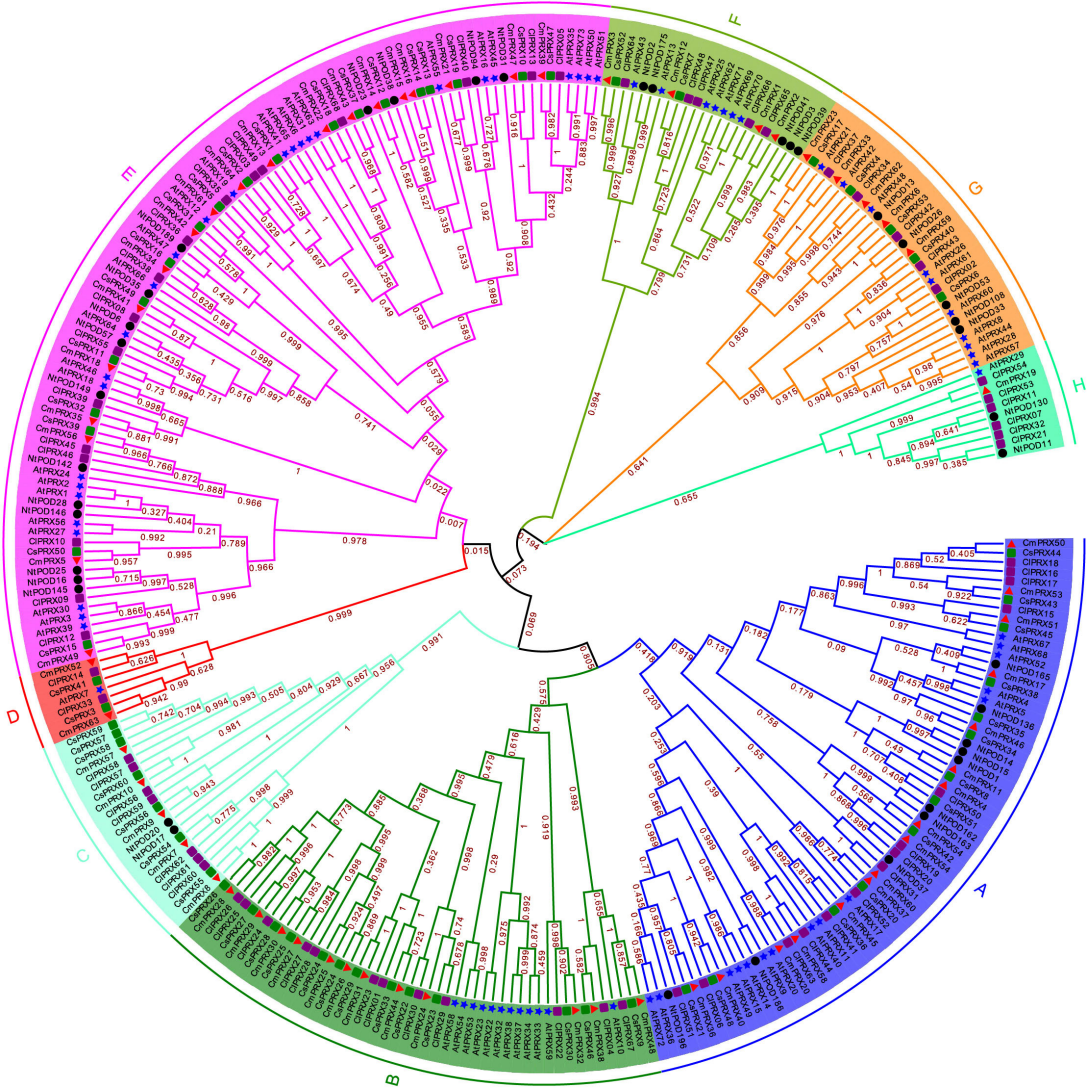
The Phylogenetic analysis of PRX proteins from melon, watermelon, cucumber, tobacco, and *Arabidopsis*. The phylogenetic tree was constructed using MEGA-X software, employing the Maximum-Likelihood method with 1000 bootstrap replicates. Each of the eight subgroups is distinguished by unique colors and letters in varying shades. Melon PRX proteins (CmPRXs) are represented by red triangles, cucumber PRXs (CsPRXs) by green squares, watermelon PRXs (ClPRXs) by purple rhombuses, *Arabidopsis* PRXs (AtPRXs) by blue stars, and tobacco PRXs (NtPODs) by black circles. Bootstrap values are indicated on the branches to provide statistical support for the phylogenetic relationships.

**Figure 3:**
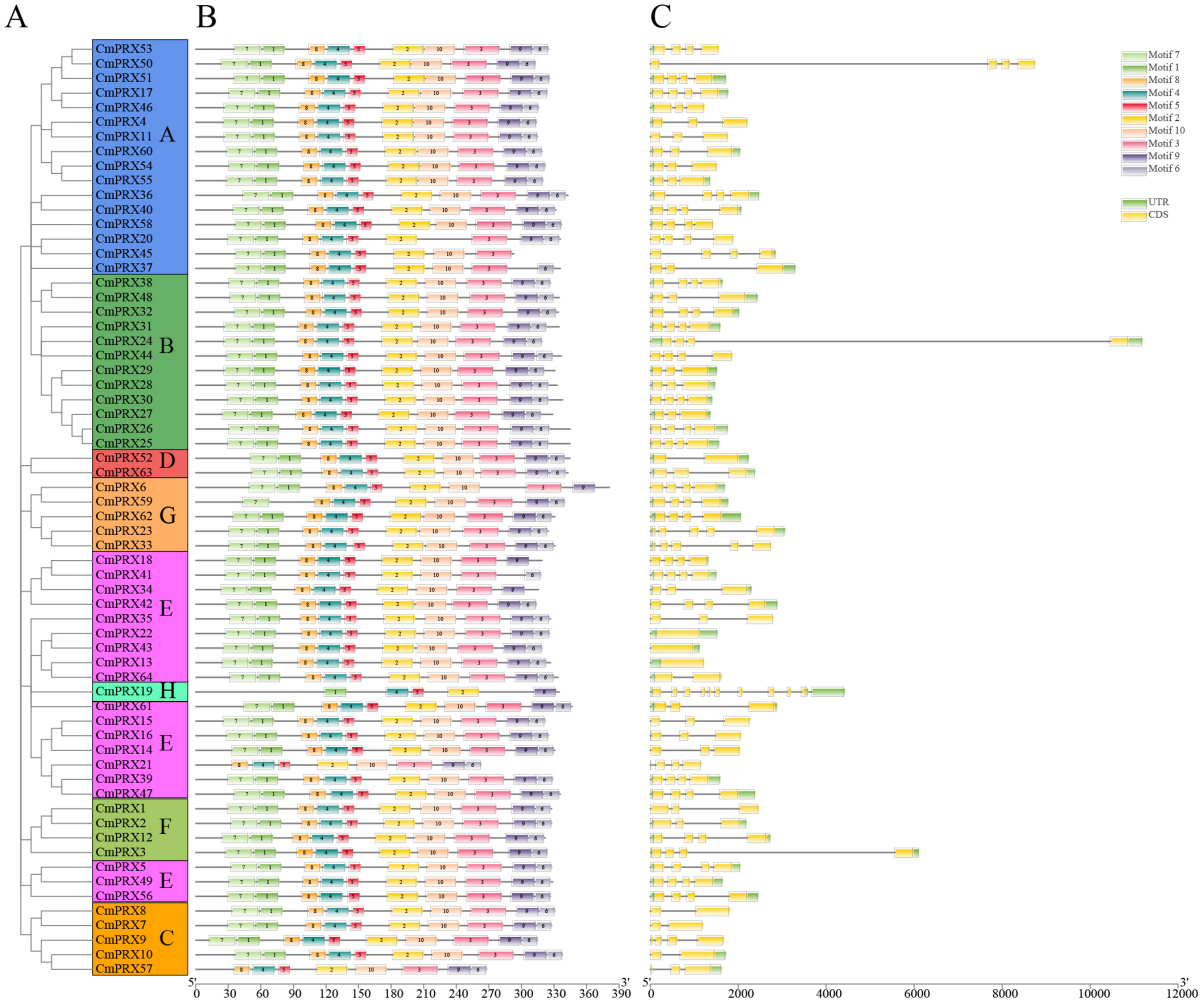
Phylogenetic relationships, protein domain architecture, and gene structure of CmPRXs. **(A)** Phylogenetic relationships among 64 CmPRX proteins were illustrated. The phylogenetic tree was generated utilizing MEGA-X software through the Maximum-Likelihood method with 1000 bootstrap replications. **(B)** Investigation of the conserved domains in CmPRX proteins. Various colors of boxes and numbers indicate distinct conserved motifs found in the CmPRX proteins. **(C)** Examination of gene structure (organization of exons and introns) of *CmPRX*s. The coding sequences (CDSs), introns, and untranslated regions (UTRs) are identified with yellow boxes, black lines, and green boxes, respectively. The scale bar is shown at the bottom.

A phylogenetic analysis of evolutionary relationships was performed on PRX proteins from three species: *Cucumis melo*, *Arabidopsis*, tobacco, cucumber, and watermelon. The analysis classified 305 PRX proteins into eight subgroups (A–H), with relatively comparable numbers of PRX members across species within the subgroups. Subgroup E contained the largest number of PRX members, comprising 19 CmPRX, 27 AtPRX, 19 CsPRX, 17 ClPRX, and 15 NtPOD proteins. This was followed by subgroup A and B, which included 16 and 12 CmPRX members, respectively. In contrast, subgroups D and H had the fewest CmPRX members, with only 2 and 1 proteins, respectively (**Figures 2**, **3A**).

### Analysis of conserved motifs and gene structure of melon PRX proteins

Conserved motif analysis of proteins showed that 53 CmPRX proteins possessed the motif7-1-8-4-3-5-2-10-3-9–6 structure, whereas the remaining 11 CmPRX proteins exhibited modifications of this pattern through either insertions or deletions. Nevertheless, all CmPRX proteins consistently harbored the fixed motif4-5–2 motif (**Figure 3B**, **Supplementary Table S2**). These findings suggested that PRX proteins are evolutionarily conserved.

Gene structure analysis demonstrated that, with the exception of *CmPRX19* containing 9 introns, the remaining *CmPRX* genes carried 0–4 introns. Specifically, 20 *CmPRX* genes possessed 2 introns, while 33 *CmPRX* genes contained 3 introns (**Figure 3C**, **Supplementary Table S2**).

### Chromosomal localization and synteny analysis of *CmPRX* genes

**Figure 4:**
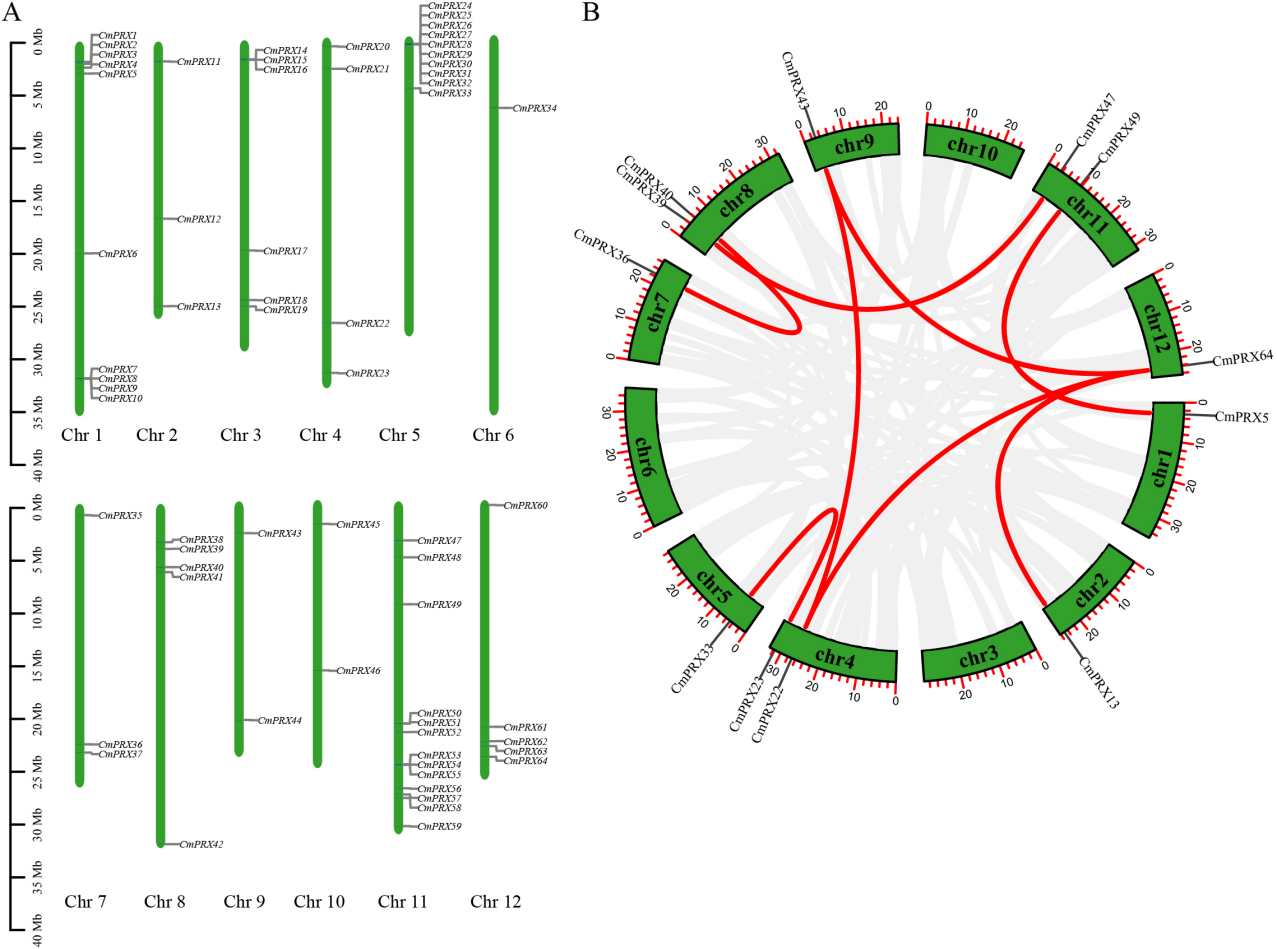
Chromosomal distribution and synteny analysis of *CmPRX* genes. **(A)** Chromosomal locations of *CmPRX* genes. Chromosome numbers are presented at the bottom of each chromosome. The numbers on the left side of the picture indicate the lengths of the chromosomes. **(B)** Synteny relationships of the *CmPRX* gene family. Gray lines present the synteny blocks in the melon genome, whereas red lines between *CmPRX* genes present the duplication events that occurred in the *CmPRX* gene family.

Chromosomal localization analysis demonstrated that the 64 *CmPRX* genes were distributed across all 12 chromosomes, with Chr 11 harboring the largest numbers, containing 13 genes. Chr 1 and Chr 5 both contained 10 genes, and Chr 6 carried only one gene (*CmPRX34*), whereas the remaining chromosomes possessed between 2 and 6 *CmPRX* genes each (**Figure 4A**). Gene duplication constitutes a key characteristic of plant genomes, and the duplication events of *CmPRX* genes in melon genome were examined. A total of eight duplicated *CmPRX* gene pairs were identified: *CmPRX5/CmPRX49*, *CmPRX64/CmPRX13*, *CmPRX64/CmPRX22*, *CmPRX22/CmPRX43*, *CmPRX23/CmPRX33*, *CmPRX36/CmPRX40*, *CmPRX47/CmPRX39*, and *CmPRX64/CmPRX43* (**Figure 4B**).

**Figure 5:**
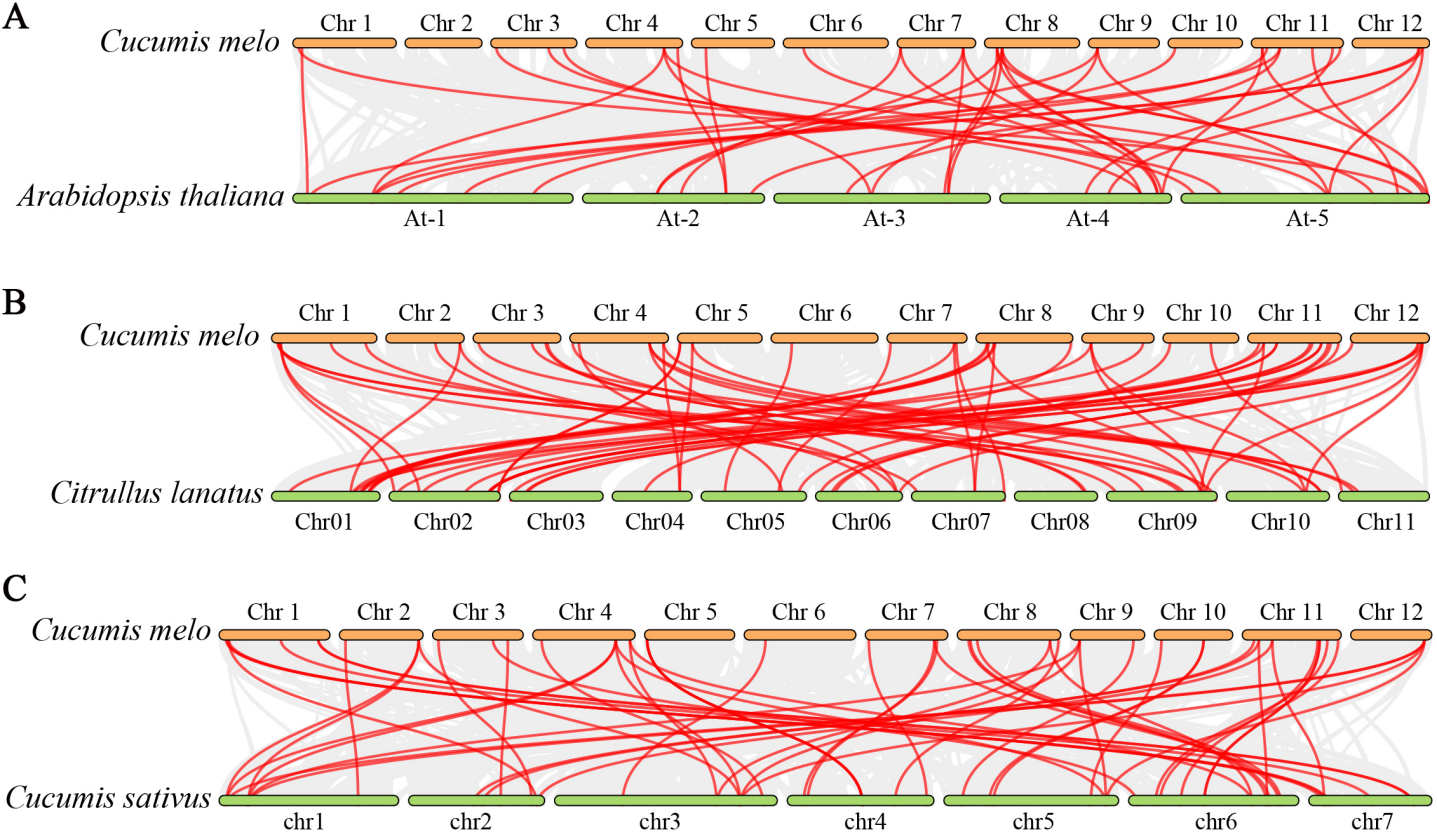
Synteny analyses of PRX genes were conducted between Cucumis melo and three other representative plant species (*Arabidopsis thaliana*, *Citrullus lanatus*, and *Cucumis sativus*). **(A)** Comparison between *C. melo* and *A. thaliana*. **(B)** Comparison between *C. melo* and *C. lanatus*. **(C)** Comparison between *C. melo* and *C. sativus*. Gray lines indicate significantly collinear blocks among the genomes of these plant species, while red lines highlight syntenic *PRX* gene pairs. The chromosome numbers are indicated at the top or bottom of each chromosome.

To elucidate the phylogenetic relationships of the *PRX* genes among various species, we constructed comparative synteny maps for three related genomes: *C. melo* versus *A. thaliana*, *C. melo* versus *Citrullus lanatus*, and *C. melo* versus *Cucumis sativus*. A total of 25 *CmPRX* genes demonstrated a syntenic relationship with genes in *Arabidopsis*, resulting in 40 pairs of syntenic genes (**Figure 5A**). Similarly, 46 *CmPRX* genes exhibited a syntenic relationship with genes in *C. lanatus*, leading to 62 pairs of syntenic genes (**Figure 5B**). Additionally, 39 *CmPRX* genes showed a syntenic relationship with genes in *C. sativus*, yielding 52 pairs of syntenic genes (**Figure 5C**) (**Supplementary Table S3**). Notably, the number of collinear gene pairs between melon and other members of the Cucurbitaceae family (cucumber and watermelon) was greater than that observed with the more distantly related *Arabidopsis*.

### Analysis of *cis*-acting elements in *CmPRX* gene promoter regions

**Figure 6:**
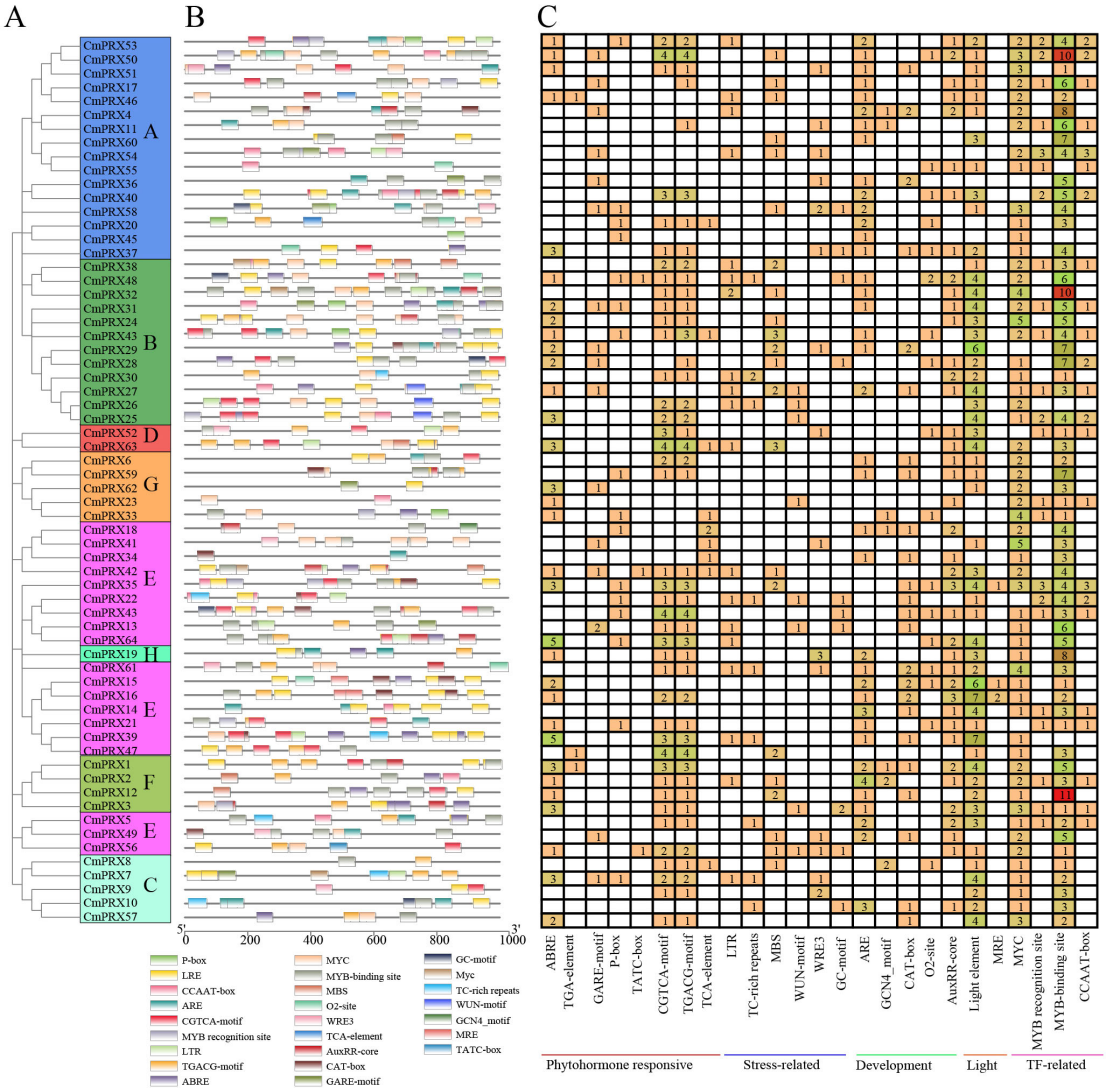
*Cis*-element analysis of the promoter regions of *CmPRX* genes. **(A)** Phylogenetic analysis of CmPRX proteins. **(B)** Different *cis*-element types and their locations in each *CmPRX* gene are indicated using colored blocks. **(C)** The numbers of different promoter elements in *CmPRX* genes are represented using different colors and numbers. PlantCARE was used to deduce the numbers, types, and locations of the potential elements in the 2 kb upstream sequence of *CmPRX* genes.

*Cis*-acting element analysis of promoter regions demonstrated that 25 categories of elements associated with hormones, stress responses, and growth and development were identified in the promoters of 64 *CmPRX* genes, exhibiting variation in both distribution and abundance (**Figure 6**, **Supplementary Table S4**). Hormone-related elements primarily comprised abscisic acid response elements (ABRE), auxin response elements (TGA-element), salicylic acid response elements (TCA-element), gibberellin response elements (P-box/TATC-box/GARE-motif), and methyl jasmonate response elements (CGTCA-motif/TGACG-motif) (**Figure 6B**, **Supplementary Table S4**). Methyl jasmonate response elements were detected in the promoter regions of 42 *CmPRX* genes, whereas only three promoters (*CmPRX1*, *CmPRX46*, and *CmPRX47*) contained TGA-elements. Stress-related elements were largely represented by motifs associated with low temperature, drought, and wounding. Among these, 19 *CmPRX* promoters carried low temperature response elements, and 22 contained drought response elements. Additionally, MYC and MYB *cis*-acting elements were identified in 57 and 60 *CmPRX* promoters, respectively (**Figure 6C**, **Supplementary Table S4**).

### Expression analysis of *CmPRX* genes

**Figure 7:**
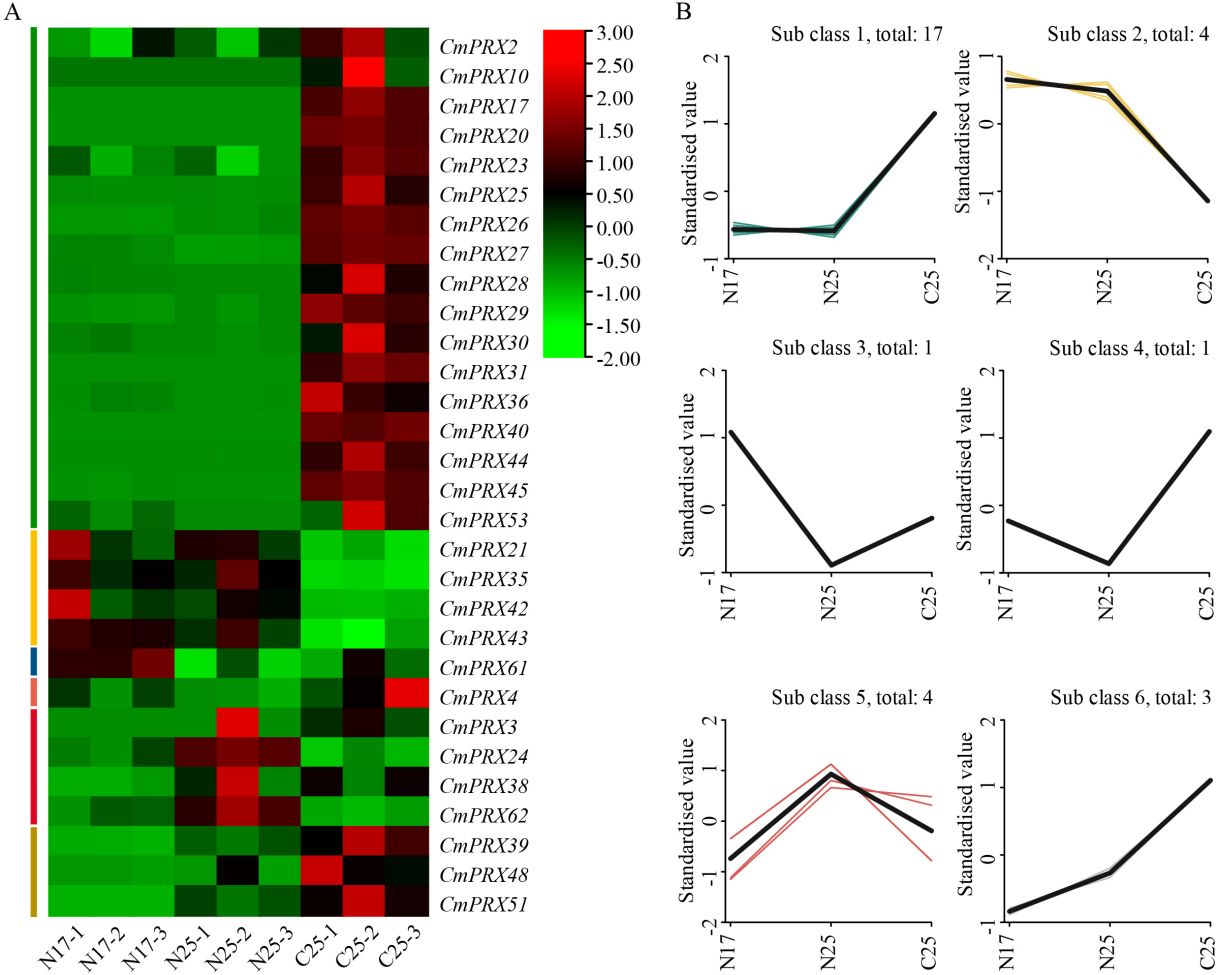
RNA-seq analysis of *CmPRX* gene expression. **(A)** Heatmap of expression levels in different samples. Red indicates higher expression of the gene in the material and green indicates lower expression. **(B)***K*-means analysis of *CmPRX* gene expression levels in different samples. N17: non-cracked fruit rind of ‘Xizhoumi 17’; N25: non-cracked fruit rind of ‘Xizhoumi 25’; C25: cracked fruit rind of ‘Xizhoumi 25’.

**Figure 8:**
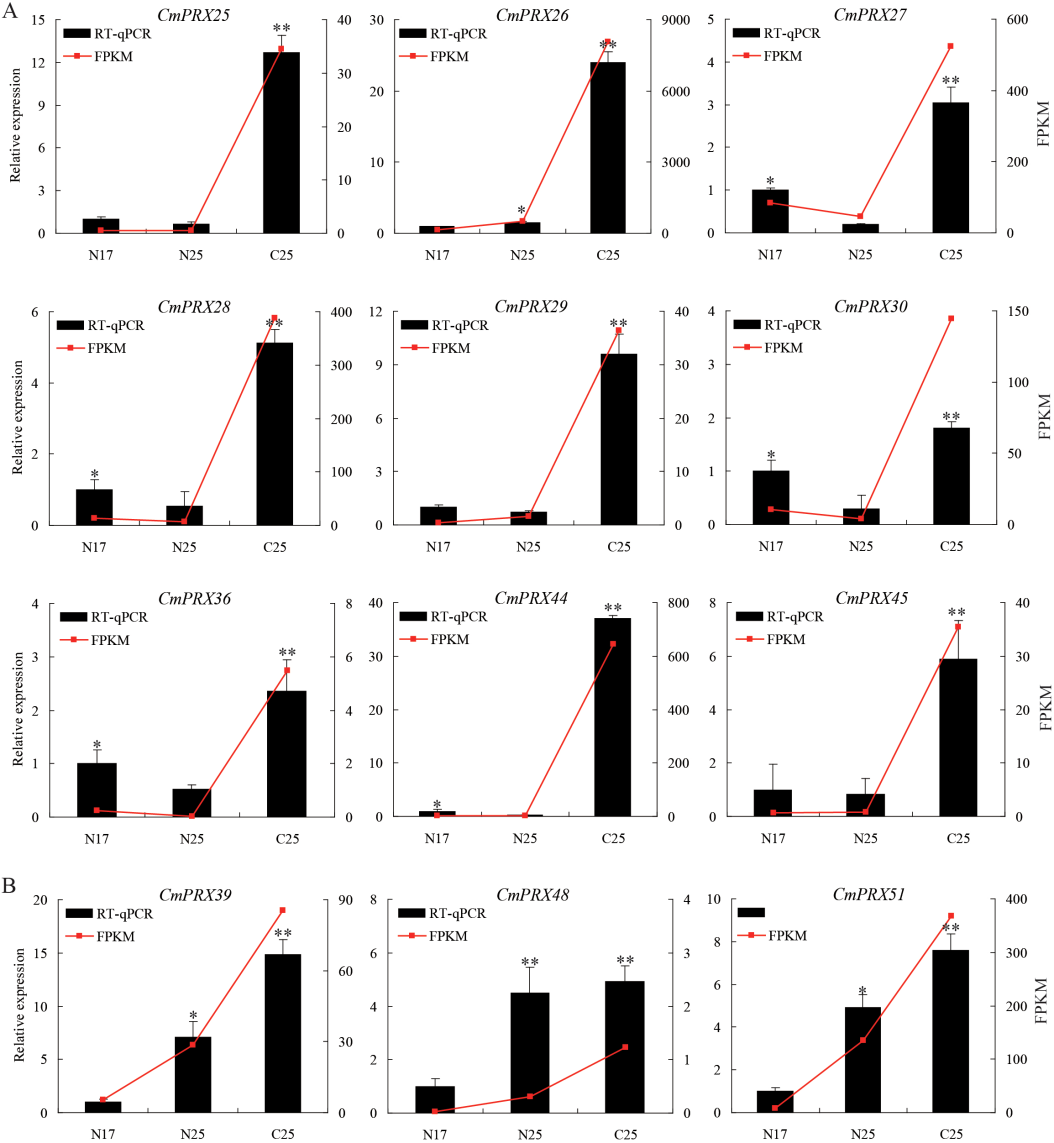
RT-qPCR validation of *CmPRX* genes in Subclass 1 **(A)** and Subclass 6 **(B)** groups. The left vertical axis represents the expression level in RT-qPCR, indicated by black bar graphs; the right vertical axis represents the expression level in RNA-seq, indicated by red lines. N17: non-cracked fruit rind of ‘Xizhoumi 17’; N25: non-cracked fruit rind of ‘Xizhoumi 25’; C25: cracked fruit rind of ‘Xizhoumi 25’. Significant differences are marked by asterisks (* and **), corresponding to *P* values of less than 0.05 and less than 0.01, respectively.

Transcriptomic data were employed to investigate the expression profiles of *CmPRX* genes. From the melon rind transcriptome, 30 *CmPRX* genes were identified, displaying distinct expression patterns among different rinds (**Figure 7A**). *K*-means clustering classified these genes into six groups (**Figure 7B**). Within these, genes belonging to Subclass 1 and Subclass 6 exhibited markedly higher expression levels in cracking-susceptible rinds (C25) compared with cracking-resistant rinds (N17, N25). RT-qPCR assays were subsequently performed for validation, and the resulting expression trends were consistent with RNA-seq data (**Figures 8A, B**). Both RT-qPCR and RNA-seq analyses demonstrated that three genes in Subclass 6 (*CmPRX39*, *CmPRX48*, *CmPRX51*) displayed a pronounced upward trajectory in expression levels across N17, N25, and C25 (**Figure 8B**), suggesting that these genes are functionally associated with rind cracking in melon.

### Expression analysis of *CmPRX* genes in different melon germplasms

**Figure 9:**
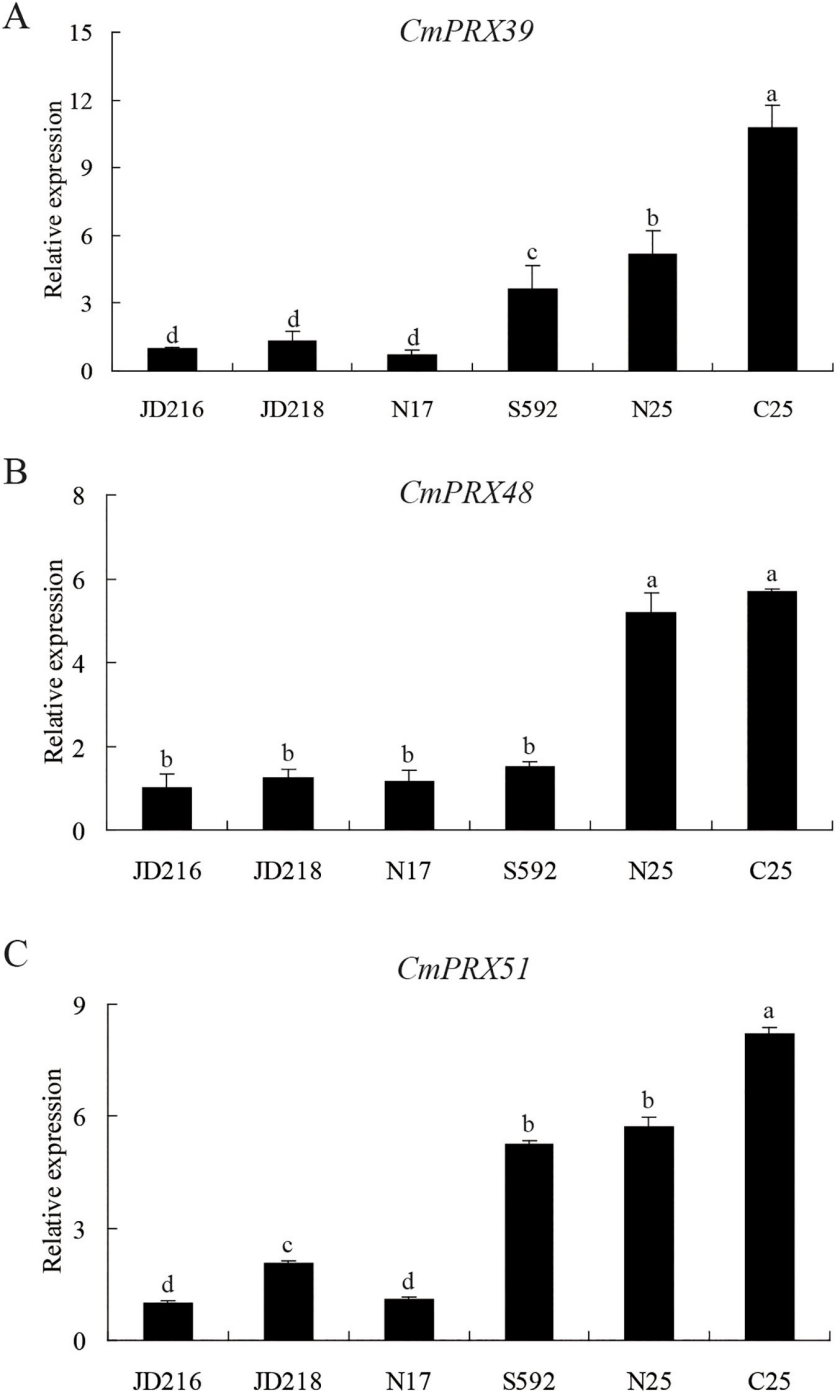
Expression analysis of three *CmPRX* genes in different melon germplasms. **(A)** Expression analysis of *CmPRX39* in different melon rinds. **(B)** Expression analysis of *CmPRX48* in different melon rinds. **(C)** Expression analysis of *CmPRX51* in different melon rinds. The x-axis displays various melon germplasms, where N17 refers to the uncracked rind of XZ17, N25 denotes the uncracked rind of XZ25, and C25 signifies the cracked rind of XZ25. Samples marked with different lowercase letters indicate statistically significant differences at the *P* < 0.05 level.

To further clarify the association between the three identified genes and fruit cracking in melon, rinds representing distinct cracking types were collected, and their expression profiles were evaluated through RT-qPCR analysis. The obtained results demonstrated that the three genes were expressed at elevated levels in cracking-susceptible rinds, whereas reduced expression was observed in the rinds of cracking-resistant germplasms (**Figures 9A-C**). Collectively, these findings suggested that *CmPRX39*, *CmPRX48*, and *CmPRX51* are functionally implicated in the regulation of rind cracking in melon.

### Prediction of the interaction network of CmPRX protein

**Figure 10:**
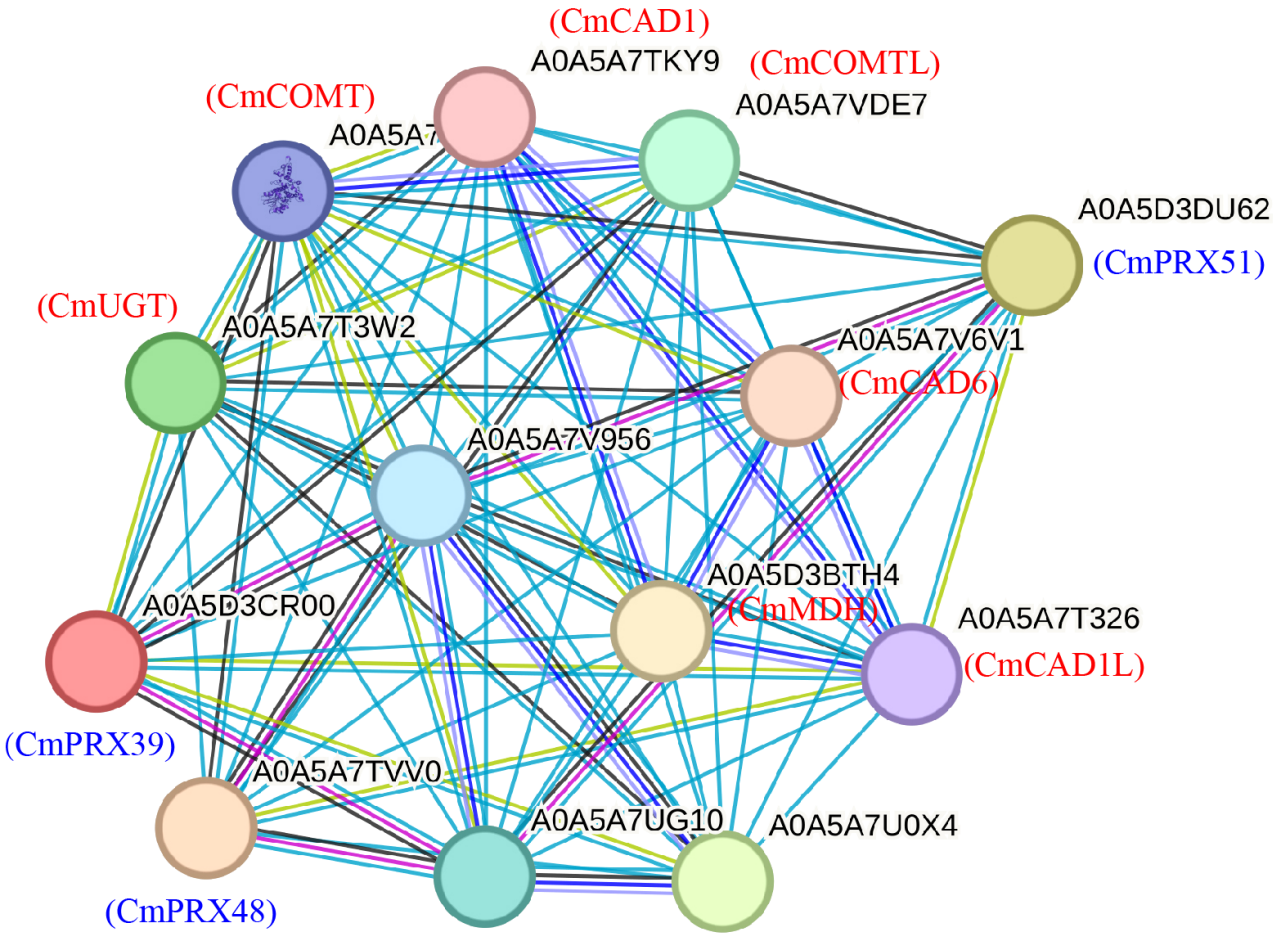
Interaction network of melon PRX proteins. This chart was constructed using three CmPRX proteins: CmPRX39, CmPRX48, and CmPRX51, which were queried in the STRING database as described in the ‘Materials and Methods’ section. Each node in the network represents all proteins produced by a single, protein-coding gene locus. The colored nodes signify the query proteins and the first shell of interactors, while the white nodes denote the second shell of interactors. Empty nodes indicate proteins with unknown three-dimensional (3D) structures, whereas filled nodes represent proteins with known or predicted 3D structures. Edges in the network illustrate protein-protein associations. The colored lines connecting the nodes indicate the type of interaction evidence: light blue lines represent known interactions from curated databases, and purple lines denote experimentally determined interactions. Predicted interactions are represented by green (gene neighborhood), red (gene fusions), and blue lines (gene co-occurrence). Additionally, yellow, black, and cyan lines represent text mining, co-expression, and protein homology, respectively.

To better understand the interaction network of CmPRX proteins and their associated proteins, we utilized STRING software to construct a comprehensive network map of selected CmPRX proteins, including CmPRX39, CmPRX48, and CmPRX51, in order to predict their functional and physical interactions. A total of 13 proteins were predicted within the network (**Figure 10**), with detailed information provided in **Supplementary Table S5**. Notably, many of these interacting proteins are implicated in the lignin synthesis pathway, such as CmCAD1, CmCAD1L, CmCAD6, CmCOMT, and CmCOMTL.

## Discussion

Plant *PRX* genes constitute a multigene family, the size and functional diversity of which vary substantially among species. In this study, 64 *CmPRX* genes were identified from the melon genome. This number is comparable to that reported in *Arabidopsis* (73 genes) (Tognolli et al., 2002) and watermelon (79 genes) (Yang et al., 2022), yet markedly lower than in maize (119 genes) (Wang et al., 2015) and rice (138 genes) (Passardi et al., 2004). Such interspecific differences in family size are likely attributable to genome duplication events, segmental gene losses, and subsequent functional diversification during evolutionary processes (Passardi et al., 2004). Moreover, the PRX gene family was found to exhibit distinct duplication patterns between monocotyledonous and dicotyledonous plants (Wang et al., 2015). Phylogenetic analysis classified the PRX proteins into eight distinct subgroups (**Figure 2**). The proportion of genes across species was consistent within each subgroup. The E subgroup contained the highest number of members among all species, followed by the A and B subgroups, while the D and H subgroups had the fewest members. This pattern suggests that the diversification of the PRX family occurred prior to species differentiation, and that its core function has remained stable throughout the evolutionary process.

The analyses of conserved motifs and gene structures further confirmed the evolutionary conservation of the *CmPRX* family, as proteins within the same subgroup generally exhibited comparable motif compositions and gene architectures (**Figure 3**). Among the 64 identified members, 53 CmPRX proteins were characterized by the complete motif7-1-8-4-5-2-10-3-9–6 tandem arrangement, while all members consistently possessed the core motif4-5-2 (**Figure 3B**). These conserved motifs were shown to encompass the PRX catalytic active centers, including the heme-binding and substrate-binding sites, which represent fundamental structural determinants required for hydrogen peroxide–dependent enzymatic reactions (Buffard et al., 1990; Welinder et al., 2002; Yang et al., 2022). With respect to gene architecture, all *CmPRX* genes contained 0–4 introns, except for *CmPRX19*, which harbored 9 introns. Furthermore, 43 of the 64 genes (67.2%) carried 2–3 introns (**Figure 3C**), a distribution consistent with the structural features of PRX genes in watermelon (Yang et al., 2022), the majority of which possess 0–3 introns. The conservation of intron number was presumed to contribute to the stability of *CmPRX* transcriptional efficiency, whereas deviations in a limited number of genes were likely associated with functional divergence resulting from insertion or deletion events during evolutionary history (Yang et al., 2010).

Chromosomal localization analysis demonstrated that 64 *CmPRX* genes were unevenly distributed across the 12 melon chromosomes. Among these, Chr 11, Chr 1, and Chr 5 represented gene-enriched regions, whereas Chr 6 was found to contain only a single gene (**Figure 4A**). This uneven distribution pattern has been frequently observed in PRX families of other plant species. In watermelon, for instance, Chr 2 harbored 22 *ClPRX* genes, while Chr 4 and Chr 10 each carried only two *ClPRX* genes, with a clustered distribution detected on Chr 1, 2, 6, 9, and 11 (Yang et al., 2022). Such heterogeneity in chromosomal allocation may be attributable to segmental duplication or tandem repeat events. Collinearity analysis identified eight pairs of duplicated *CmPRX* genes, all of which originated from segmental duplication (**Figure 4B**). In watermelon, 12 pairs of segmental duplication genes and 25 pairs of tandem duplication genes were identified (Yang et al., 2022). In maize, both segmental and tandem duplications contributed to *PRX* gene expansion (Wang et al., 2015), whereas in rice (Passardi et al., 2004) and Chinese pear (Cao et al., 2016), segmental duplication was primarily responsible for this expansion. These findings suggested that segmental duplication events likely represent the predominant driving force behind *CmPRX* gene expansion and subsequent functional divergence, while interspecific differences in gene copy number and subgroup partitioning may be associated with gene loss and lineage-specific expansion. Additionally, the higher number of collinear gene pairs between melon and other Cucurbitaceae species, such as watermelon and cucumber, compared to *Arabidopsis* (**Figure 5**), aligns with their closer phylogenetic relationship. This observation reflects the conservation of the PRX gene family throughout the evolution of Cucurbitaceae. Furthermore, this syntenic conservation suggests that these orthologous *PRX* genes may share similar biological functions, thereby providing a valuable reference for inferring the functions of *CmPRX* genes based on their orthologs in other well-studied plants.

Functional divergence or redundancy commonly arises following gene duplication. In this study, *CmPRX64* was identified as having duplication relationships with *CmPRX13*, *CmPRX22*, and *CmPRX43* (**Figure 4B**). The promoters of these genes were found to contain MeJA-responsive elements (CGTCA-motif) as well as MYB binding sites (**Figure 6C**, **Supplementary Table S4**), indicating that the duplicated members may retain redundant functions and act jointly in MeJA-mediated biological processes. A similar phenomenon has been documented in *Arabidopsis*, where *PRX9* and *PRX40* are duplicated genes that together preserve tapetal cell wall integrity (Jacobowitz et al., 2019), thereby reinforcing the concept of functional redundancy among duplicated genes.

Promoter *cis*-acting elements interact with specific transcription factors and function as essential regulatory sites for gene transcription, with their types and abundances determining the specificity of gene responses to environmental and hormonal cues (Lee and Young, 2013). In this study, 25 categories of *cis*-acting elements were identified in the promoters of 64 *CmPRX* genes. Among these, hormone-responsive and stress-responsive elements accounted for the largest proportions, indicating that *CmPRX* genes may participate in the regulation of melon fruit cracking through the integration of hormonal and stress signaling pathways. Within the hormone-responsive group, CGTCA-motif/TGACG-motif were the most prevalent, present in 42 *CmPRX* gene promoter regions, substantially exceeding the numbers of abscisic acid–responsive elements (28), TCA-elements (15), and TGA-elements (3). MeJA is recognized as a key regulator of plant cell wall metabolism and stress adaptation. Application of exogenous jasmonic acid has been shown to increase fruit dehiscence and slow down softening rates in apple (Rudell et al., 2005). Recent studies have further demonstrated that the JA content in the exocarp and mesocarp of cracking-susceptible jujube varieties was higher than that in resistant varieties at both the half-red and full-red ripening stages. After exogenous MeJA treatment, the cracking index of jujube fruits was significantly elevated, accompanied by strong enrichment of genes involved in cell wall metabolism (Liu et al., 2023). In this study, most *CmPRX* genes exhibited elevated expression in cracked rinds compared with non-cracked rinds (**Figures 7**, **8**). It is hypothesized that MeJA promotes fruit cracking by inducing *CmPRX* gene expression.

The mechanical properties of the cell wall and structural changes in the exocarp are critical determinants of fruit rind cracking. Lignin deposition increases cell wall rigidity and reduces extensibility, thereby decreasing the mechanical tolerance of the pericarp to internal turgor pressure (Bruggenwirth and Knoche, 2017). Histological observations revealed that the exocarp cells of the ‘XZ25’ variety, known for its susceptibility to cracking, were arranged loosely and in a disorderly manner. In contrast, the pericarp cells of the ‘XZ17’ variety, which exhibits resistance to cracking, were organized closely and in an orderly fashion (Hu et al., 2025). Our study found that there was a greater accumulation of lignin in the rind of ‘XZ25’ (**Figure 1D**), which may directly compromise the exocarp’s ability to withstand expansion stress. POD functions as the principal enzyme catalyzing the polymerization of lignin monomers (Barros et al., 2015), and elevated POD activity accelerates lignin biosynthesis. In tomato, POD activity has been reported to be markedly higher in cracking-susceptible varieties compared with resistant ones, and its levels in cracked fruits were greater than in non-cracked fruits (Yang et al., 2016; Zhang et al., 2020). This phenomenon is likely related to the role of POD in mediating the cross-linking of phenolic compounds within cell walls, which enhances POD activity, subsequently reinforcing wall rigidity and reducing the mechanical flexibility of pericarp tissues (Elstner, 1982; Zhu et al., 2020). In this study, both POD activity and lignin content were significantly higher in cracked rinds of ‘XZ25’ (C25) compared with non-cracked rinds (N25), and overall levels in cracking-susceptible varieties exceeded those in resistant varieties (**Figure 1**). These findings were in strong agreement with previous reports. Furthermore, RNA-seq and RT-qPCR analyses revealed that three genes in Subclass 6 (*CmPRX39*, *CmPRX48*, *CmPRX51*) exhibited markedly elevated expression in C25 relative to N17 and N25 (**Figure 8B**). Additionally, the expression profiles of these three genes were consistent across different melon germplasms, showing a sequential decline from ‘XZ25’ (cracking-susceptible) to ‘S592’ (moderately cracking) and ‘XZ17’ (cracking-resistant), thereby suggesting that these genes represent key candidates involved in regulating rind cracking in melon. Prediction of protein-protein interaction networks indicated that the three candidate genes interacted with 13 proteins, several of which are directly involved in the lignin synthesis pathway, including CmCAD1, CmCAD1L, CmCAD6, CmCOMT, and CmCOMTL (**Figure 10**). CAD and COMT are pivotal enzymes in the biosynthesis of lignin monomers: CAD catalyzes the final step of lignin monomer formation by reducing cinnamaldehydes to cinnamyl alcohols, while COMT is involved in the methylation of lignin precursors, leading to the formation of G-lignin and S-lignin monomers (Kumar et al., 2016). The interactions between CmPRX39, CmPRX48, and CmPRX51 with these lignin synthesis-related enzymes strongly support the hypothesis that these CmPRXs regulate melon rind cracking by mediating lignin polymerization. This protein-protein interaction network establishes a direct molecular link between CmPRXs and the lignin synthesis pathway, further validating their functional role in rind cracking.

Further characterization of these three genes revealed that their promoter regions contained MeJA-responsive elements (CGTCA-motif/TGACG-motif) (**Figure 6**, **Supplementary Table S4**), and our unpublished data confirmed that MeJA content in cracking-susceptible melon rinds is significantly higher than in resistant varieties. This suggests a MeJA-CmPRX-lignin regulatory module: exogenous or endogenous MeJA induces the expression of *CmPRX* genes, accelerates lignin polymerization, enhances cell wall rigidity, and ultimately promotes rind cracking. Abscisic acid (ABA) may also be involved, as ABRE elements were detected in their promoters—ABA has been reported to regulate cell wall metabolism and stress responses in fruit (Liu et al., 2023), and its interaction with MeJA in mediating *CmPRX* expression warrants further investigation. *CmPRX39* and *CmPRX47* exhibited a duplication relationship (**Figure 4B**), with *CmPRX39* showing high expression in cracked rinds, whereas *CmPRX47* was not detected in RNA-seq data (**Figure 6A**), implying possible functional redundancy between them. CmPRX51 clusters with *Arabidopsis* PRX9 (**Figure 2**), which is involved in cell wall integrity maintenance (Jacobowitz et al., 2019). It is hypothesized that *CmPRX39/48/51* may promote melon rind cracking by enhancing lignin polymerization and reinforcing cell wall rigidity. To further confirm the functional roles of *CmPRX39*, *CmPRX48*, and *CmPRX51* in melon rind cracking, we plan to conduct the following follow-up experiments: (1) Virus-induced gene silencing (VIGS) will be employed to silence these three genes in cracking-susceptible ‘XZ25’ plants. Changes in the rind cracking rate, lignin content, and peroxidase (POD) activity will be measured to verify whether gene silencing reduces susceptibility to cracking; (2) Overexpression vectors of the candidate genes will be constructed and transformed into cracking-resistant ‘XZ17’ via *Agrobacterium*-mediated transformation. The phenotypic and physiological changes of the transgenic plants will be observed to confirm whether gene overexpression promotes rind cracking; (3) CRISPR-Cas9 technology will be utilized to generate knockout mutants of the candidate genes in ‘XZ25’, and the stability of the cracking-resistant phenotype will be evaluated. These functional validation experiments will provide direct evidence for the regulatory roles of *CmPRX39*, *CmPRX48*, and *CmPRX51* in melon rind cracking.

## Conclusion

This study demonstrated that POD activity and lignin content in the rinds of cracking-susceptible melon were significantly higher than those in resistant varieties, and both parameters showed strong positive correlations with the rind cracking rate. These findings confirm that POD-mediated lignin polymerization represents the central physiological mechanism regulating rind cracking in melon. Sixty-four *CmPRX* genes were identified from the melon genome, which were found to be highly conserved with PRX proteins from *Arabidopsis*, watermelon, cucumber, and tobacco. Collinearity analysis revealed eight segmentally duplicated *CmPRX* gene pairs within the melon genome, and comparative synteny analysis showed that melon shares more collinear *PRX* gene pairs with other Cucurbitaceae species (watermelon and cucumber) than with *Arabidopsis*, reflecting the evolutionary conservation of the *PRX* gene family in Cucurbitaceae. The *CmPRX* genes were unevenly distributed across 12 chromosomes, and their promoters contained abundant hormone-responsive elements (predominantly MeJA) as well as stress-responsive elements, suggesting potential involvement in regulating melon rind physiology through the integration of multiple signaling pathways. Transcriptome analysis combined with RT-qPCR validation further revealed that *CmPRX39*, *CmPRX48*, and *CmPRX51* were specifically and highly expressed in cracked rinds. Protein interaction network prediction showed that these three candidate genes interact with multiple key enzymes in the lignin synthesis pathway (e.g., CmCAD1, CmCOMT), providing a direct molecular link between *CmPRX* genes and lignin polymerization. Collectively, these results identify *CmPRX39*, *CmPRX48*, and *CmPRX51* as key candidate genes implicated in the regulation of rind cracking in melon, offering valuable molecular targets for the genetic improvement of cracking-resistant melon cultivars.

## Data Availability

The data presented in the study are deposited in the NCBI repository under SRA accession number: SRP466450.

## References

[B1] Almagro L. Gomez Ros L. V. Belchi-navarro S. Bru R. Ros Barcelo A. Pedreno M. A. (2009). Class III peroxidases in plant defence reactions. J. Exp. Bot. 60, 377–390. doi: 10.1093/jxb/ern277, PMID: 19073963

[B2] Barros J. Serk H. Granlund I. Pesquet E. (2015). The cell biology of lignification in higher plants. Ann. Bot. 115, 1053–1074. doi: 10.1093/aob/mcv046, PMID: 25878140 PMC4648457

[B3] Bruggenwirth M. Knoche M. (2017). Cell wall swelling, fracture mode, and the mechanical properties of cherry fruit skins are closely related. Planta 245, 765–777. doi: 10.1007/s00425-016-2639-7, PMID: 28012001

[B4] Buffard D. Breda C. van Huystee R. B. Asemota O. Pierre M. Ha D. B. . (1990). Molecular cloning of complementary DNAs encoding two cationic peroxidases from cultivated peanut cells. Proc. Natl. Acad. Sci. U.S.A. 87, 8874–8878. doi: 10.1073/pnas.87.22.8874, PMID: 2247460 PMC55062

[B5] Cao Y. P. Han Y. H. Meng D. D. Li D. Jin Q. Lin Y. . (2016). Structural, evolutionary, and functional analysis of the Class III peroxidase gene family in Chinese pear (*Pyrus bretschneideri*). Front. Plant Sci. 7. doi: 10.3389/fpls.2016.01874, PMID: 28018406 PMC5145892

[B6] Chen C. Chen H. Zhang Y. Thomas H. R. Frank M. H. He Y. . (2020). TBtools: An integrative toolkit developed for interactive analyses of big biological data. Mol. Plant 13, 1194–1202. doi: 10.1016/j.molp.2020.06.009, PMID: 32585190

[B7] Cheng L. Ma L. Meng L. Shang H. Cao P. Jin J. (2022). Genome-wide identification and analysis of the class III peroxidase gene family in tobacco (*Nicotiana tabacum*). Front. Genet. 13. doi: 10.3389/fgene.2022.916867, PMID: 35769995 PMC9234461

[B8] Correia S. Schouten R. Silva A. P. Goncalves B. (2018). Sweet cherry fruit cracking mechanisms and prevention strategies: A review. Sci. Hortic. 240, 369–377. doi: 10.1016/j.scienta.2018.06.042

[B9] Elstner E. F. (1982). Oxygen activation and oxygen toxicity. Annu. Rev. Plant Physiol. 33, 73–96. doi: 10.1146/annurev.pp.33.060182.000445

[B10] Fan R. Liu B. Duan X. Li M. Zhang Y. Zhang X. . (2025). Cytological, phytohormone, and transcriptomic analyses reveal the key genes and pathways involved in melon fruit cracking. Horticulturae 11, 227. doi: 10.3390/horticulturae11030227

[B11] Fan R. Yang Y. Li M. Zhang Y. Yi H. (2023). Analysis of cracking resistance and physiological characteristic on Xinjiang muskmelon. Acta Bot. Boreal.-Occident. Sin. 43, 1146–1157.

[B12] Hu Y. Li Y. Zhang T. Wang C. Zhu B. Tian L. . (2025). Integrated morphological, physicochemical, metabolomic, and transcriptomic analyses elucidate the mechanism underlying melon (*Cucumis melo* L.) peel cracking. Agriculture 15, 2475. doi: 10.3390/agriculture15232475

[B13] Hu Y. Li Y. Zhu B. Huang W. Chen J. Wang F. . (2024). Genome-wide identification of the expansin gene family in netted melon and their transcriptional responses to fruit peel cracking. Front. Plant Sci. 15. doi: 10.3389/fpls.2024.1332240, PMID: 38322822 PMC10846642

[B14] Hu Y. Zhang T. Wang P. Li Y. Wang M. Zhu B. . (2023). Genome-wide characterization of HSP90 gene family in Chinese pumpkin (*Cucurbita moschata* Duch.) and their expression patterns in response to heat and cold stresses. Agronomy 13, 430. doi: 10.3390/agronomy13020430

[B15] Jacobowitz J. R. Doyle W. C. Weng J. K. (2019). PRX9 and PRX40 are extensin peroxidases essential for maintaining tapetum and microspore cell wall integrity during *Arabidopsis* anther development. Plant Cell 31, 848–861. doi: 10.1105/tpc.18.00907, PMID: 30886127 PMC6501601

[B16] Khadivi-Khub A. (2015). Physiological and genetic factors influencing fruit cracking. Acta Physiol. Plant 37, 1718. doi: 10.1007/s11738-014-1718-2

[B17] Kumar M. Campbell L. Turner S. (2016). Second cell walls: biosynthesis and manipulation. J. Exp. Bot. 67, 515–531. doi: 10.1093/jxb/erv533, PMID: 26663392

[B18] Kunieda T. Shimada T. Kondo M. Nishimura M. Nishitani K. Hara-Nishimura I. (2013). Spatiotemporal secretion of PEROXIDASE36 is required for seed coat mucilage extrusion in *Arabidopsis*. Plant Cell 25, 1355–1367. doi: 10.1105/tpc.113.110072, PMID: 23572548 PMC3663273

[B19] Lee T. I. Young R. A. (2013). Transcriptional regulation and its misregulation in disease. Cell 152, 1237–1251. doi: 10.1016/j.cell.2013.02.014, PMID: 23498934 PMC3640494

[B20] Liszkay A. van der Zalm E. Schopfer P. (2004). Production of reactive oxygen intermediates (O2.^-^, H_2_O_2_, and.OH) by maize roots and their role in wall loosening and elongation growth. Plant Physiol. 136, 3114–3123. doi: 10.1104/pp.104.044784, PMID: 15466236 PMC523372

[B21] Liu Y. L. Chen S. Y. Liu G. T. Jia X. Y. Haq S. Deng Z. J. . (2022). Morphological, physiochemical, and transcriptome analysis and CaEXP4 identification during pepper (*Capsicum annuum* L.) fruit cracking. Sci. Hortic. 297, 110982. doi: 10.1016/j.scienta.2022.110982

[B22] Liu N. Zhao H. Hou L. Zhang C. Bo W. Pang X. . (2023). HPLC-MS/MS-based and transcriptome analysis reveal the effects of ABA and MeJA on jujube (*Ziziphus jujuba* Mill.) cracking. Food Chem. 421, 136155. doi: 10.1016/j.foodchem.2023.136155, PMID: 37126870

[B23] Luo W. Liu J. Xu W. Zhi S. Wang X. Sun Y. (2024). Molecular characterization of peroxidase (*PRX*) gene family in cucumber. Genes 15, 1245. doi: 10.3390/genes15101245, PMID: 39457369 PMC11507654

[B24] Musel G. Schindler T. Bergfeld R. Ruel K. Jacquet G. Lapierre C. . (1997). Structure and distribution of lignin in primary and secondary cell walls of maize coleoptiles analyzed by chemical and immunological probes. Planta 201, 146–159. doi: 10.1007/BF01007699

[B25] Passardi F. Cosio C. Penel C. Dunand C. (2005). Peroxidases have more functions than a Swiss army knife. Plant Cell Rep. 24, 255–265. doi: 10.1007/s00299-005-0972-6, PMID: 15856234

[B26] Passardi F. Longet D. Penel C. Dunand C. (2004). The class III peroxidase multigenic family in rice and its evolution in land plants. Phytochemistry 65, 1879–1893. doi: 10.1016/j.phytochem.2004.06.023, PMID: 15279994

[B27] Qi Z. Y. Li J. X. Raza M. A. Zou X. Cao L. Rao L. . (2015). Inheritance of fruit cracking resistance of melon (*Cucumis melo* L.) fitting E-0 genetic model using major gene plus polygene inheritance analysis. Sci. Hortic. 189, 168–174. doi: 10.1016/j.scienta.2015.04.004

[B28] Rudell D. R. Fellman J. K. Mattheis J. P. (2005). Preharvest application of methyl jasmonate to ‘Fuji’ apples enhances red coloration and affects fruit size, splitting, and bitter pit incidence. HortScience 40, 1760–1762. doi: 10.21273/HORTSCI.40.6.1760

[B29] Santos M. Egea-Cortines M. Goncalves B. Matos M. (2023). Molecular mechanisms involved in fruit cracking: A review. Front. Plant Sci. 14. doi: 10.3389/fpls.2023.1130857, PMID: 36937999 PMC10016354

[B30] Schumann C. Winkler A. Brüggenwirth M. Köpcke K. Knoche M. (2019). Crack initiation and propagation in sweet cherry skin: A simple chain reaction causes the crack to ‘run’. PloS One 14, e0219794. doi: 10.1371/journal.pone.0219794, PMID: 31365556 PMC6668808

[B31] Shen T. Wang Q. Ju H. Tian R. Fu D. Bu X. . (2025). The Class III peroxidase OsPrx20 is a key regulator of stress response and growth in rice. Plant Commun. 8, 101487. doi: 10.1101/2024.04.08.588571, PMID: 40842157 PMC12546647

[B32] Simon G. (2006). Review on rain induced fruit cracking of sweet cherries (*Prunus avium* L.), its causes and the possibilities of prevention. Int. J. Hortic. Sci. 12, 27–35. doi: 10.31421/IJHS/12/3/654

[B33] Subramanian B. Gao S. Lercher M. J. Hu S. Chen W. H. (2019). Evolview v3: a webserver for visualization, annotation, and management of phylogenetic trees. Nucleic Acids Res. 47, W270–W275. doi: 10.1093/nar/gkz357, PMID: 31114888 PMC6602473

[B34] Szklarczyk D. Kirsch R. Koutrouli M. Nastou K. Mehryary F. Hachilif R. . (2023). The STRING database in 2023: protein–protein association networks and functional enrichment analyses for any sequenced genome of interest. Nucleic Acids Res. 51, D638–D646. doi: 10.1093/nar/gkac1000, PMID: 36370105 PMC9825434

[B35] Tognolli M. Penell C. Greppin H. Simon P. (2002). Analysis and expression of the class III peroxidase large gene family in *Arabidopsis thaliana*. Gene 288, 1290138. doi: 10.1016/S0378-1119(02)00465-1, PMID: 12034502

[B36] Vogelsang L. Dietz K. J. (2022). Plant thiol peroxidases as redox sensors and signal transducers in abiotic stress acclimation. Free Radical Biol. Med. 193, 764–778. doi: 10.1016/J.FREERADBIOMED.2022.11.019, PMID: 36403735

[B37] Wang J. G. Gao X. M. Ma Z. L. Chen J. Liu Y. (2019). Analysis of the molecular basis of fruit cracking susceptibility in *Litchi chinensis* cv. Baitangying by transcriptome and quantitative proteome profiling. J. Plant Physiol. 234-235, 106–116. doi: 10.1016/j.jplph.2019.01.014, PMID: 30753966

[B38] Wang Y. Guo L. Zhao X. Zhao Y. Hao Z. Luo H. . (2021). Advances in mechanisms and omics pertaining to fruit cracking in horticultural plants. Agronomy 11, 1045. doi: 10.3390/agronomy11061045

[B39] Wang Y. Wang Q. Zhao Y. Han G. Zhu S. (2015). Systematic analysis of maize class III peroxidase gene family reveals a conserved subfamily involved in abiotic stress response. Gene 566, 95–108. doi: 10.1016/j.gene.2015.04.041, PMID: 25895479

[B40] Welinder K. G. Justesen A. F. Jaersgard I. V. K. Jensen R. B. Rasmussen S. K. Jespersen H. M. . (2002). Structural diversity and transcription of Class-IIl peroxidases from *Arabidopsis thaliana*. Eur. J. Biochem. 269, 6063–6081. doi: 10.1046/j.1432-1033.2002.03311.x, PMID: 12473102

[B41] Yang L. Takuno S. Waters E. R. Gaut B. S. (2010). Lowly expressed genes in Arabidopsis thaliana bear the signature of possible pseudogenization by promoter degradation. Mol. Biol. Evol. 28, 1193–1203. doi: 10.1093/molbev/msq298, PMID: 21059790

[B42] Yang Z. Wu Z. Zhang C. Hu E. Zhou R. Jiang F. (2016). The composition of pericarp, cell aging, and changes in water absorption in two tomato genotypes: mechanism, factors, and potential role in fruit cracking. Acta Physiol. Plant 38, 215. doi: 10.1007/s11738-016-2228-1

[B43] Yang T. Zhang P. Pan J. Amanullah S. Luan F. Han W. . (2022). Genome-wide analysis of the peroxidase gene family and verification of lignin synthesis-related genes in watermelon. Int. J. Mol. Sci. 23, 642. doi: 10.3390/ijms23020642, PMID: 35054827 PMC8775647

[B44] Zhang C. Zhao Y. J. Jiang F. L. Wu Z. Cui S. Y. Lv H. M. . (2020). Differences of reactive oxygen species metabolism in top, middle and bottom part of epicarp and mesocarp influence tomato fruit cracking. J. Hortic. Sci. Biotechnol. 95, 746–756. doi: 10.1080/14620316.2020.1748525

[B45] Zhu M. T. Yu J. Zhao M. Wang M. J. Yang G. S. (2020). Transcriptome analysis of metabolisms related to fruit cracking during ripening of a cracking-susceptible grape berry cv. Xiangfei (*Vitis vinifera* L.). Genes Genom. 42, 639–650. doi: 10.1007/s13258-020-00930-y, PMID: 32274647

