# Supplementary material for: Genome-wide identification of the class III peroxidase gene family and its association with fruit rind cracking in Cucumis melo
Source: Front Plant Sci. 2026 Jan 21;16:1706618. doi: 10.3389/fpls.2025.1706618 (PMC12868172; doi:10.3389/fpls.2025.1706618)
Supplement: Supplementary file 1 [file Table1.docx]

Supplementary Material

**Genome-wide identification of the Class III peroxidase gene family and its association with fruit rind cracking in *Cucumis melo***

**Yanping Hu^1, 2, 4, †^, Tingting Zhang^3, †^, Yushan Wang^2^, Chongchong Wang^1, 4^, Baibi Zhu^1^, Feng Wang^1, 4^, Yisong Chen^1, 4^, Min Wang^1, 4, *^, Yang Zhou^2, *^**

^1^ The Institute of Vegetables, Hainan Academy of Agricultural Sciences, Key Laboratory of Vegetable Biology of Hainan Province, Hainan Vegetable Breeding Engineering Technology Research Center, Haikou, China

^2^ Key Laboratory for Quality Regulation of Tropical Horticultural Crops of Hainan Province, School of Tropical Agriculture and Forestry (School of Agricultural and Rural Affairs, School of Rural Revitalization), Hainan University, Haikou, China

^3^ Xiangyang Academy of Agricultural Sciences, Xiangyang, China

^4^ Sanya Institute, Hainan Academy of Agricultural Sciences, Sanya, China

*** Correspondence:** Yang Zhou: zhouyang@hainanu.edu.cn; Min Wang: 13005022331@163.com

**^†^** These authors have contributed equally to this work.

**Supplementary Table S1** Primers used in this study.

**Supplementary Table S2** Composition of motifs and number of introns of CmPRX gene family.

**Supplementary Table S3** The collinear gene pairs between *CmPRX* and other species.

**Supplementary Table S4** Information of *cis*-elements in the *CmPRX* promoters.

**Supplementary Table S5** Informations of the network proteins.
